# Mechanisms Underlying Metabolic and Neural Defects in Zebrafish and Human Multiple Acyl-CoA Dehydrogenase Deficiency (MADD)

**DOI:** 10.1371/journal.pone.0008329

**Published:** 2009-12-17

**Authors:** Yuanquan Song, Mary A. Selak, Corey T. Watson, Christopher Coutts, Paul C. Scherer, Jessica A. Panzer, Sarah Gibbs, Marion O. Scott, Gregory Willer, Ronald G. Gregg, Declan W. Ali, Michael J. Bennett, Rita J. Balice-Gordon

**Affiliations:** 1 Department of Neuroscience, University of Pennsylvania School of Medicine, Philadelphia, Pennsylvania, United States of America; 2 Children's Hospital of Philadelphia Research Institute, Children's Hospital of Philadelphia and University of Pennsylvania, Philadelphia, Pennsylvania, United States of America; 3 Department of Biochemistry and Molecular Biology, University of Louisville, Louisville, Kentucky, United States of America; 4 Department of Biological Sciences, University of Alberta, Edmonton, Alberta, Canada; 5 Department of Pathology and Laboratory Medicine, University of Pennsylvania School of Medicine and Children's Hospital of Philadelphia, Philadelphia, Pennsylvania, United States of America; Texas A&M University, United States of America

## Abstract

In humans, mutations in electron transfer flavoprotein (ETF) or electron transfer flavoprotein dehydrogenase (ETFDH) lead to MADD/glutaric aciduria type II, an autosomal recessively inherited disorder characterized by a broad spectrum of devastating neurological, systemic and metabolic symptoms. We show that a zebrafish mutant in ETFDH, *xavier*, and fibroblast cells from MADD patients demonstrate similar mitochondrial and metabolic abnormalities, including reduced oxidative phosphorylation, increased aerobic glycolysis, and upregulation of the PPARG-ERK pathway. This metabolic dysfunction is associated with aberrant neural proliferation in *xav*, in addition to other neural phenotypes and paralysis. Strikingly, a PPARG antagonist attenuates aberrant neural proliferation and alleviates paralysis in *xav*, while PPARG agonists increase neural proliferation in wild type embryos. These results show that mitochondrial dysfunction, leading to an increase in aerobic glycolysis, affects neurogenesis through the PPARG-ERK pathway, a potential target for therapeutic intervention.

## Introduction

Mitochondria, the cellular power plants in most eukaryotic organisms, play pivotal roles in cell signaling, differentiation, and the control of cell cycle, growth and death. Particularly in the nervous system, mitochondrial function is essential in meeting the high energy demand in neurons and glia[1], [2]. During nervous system development, mitochondria regulate neural proliferation and differentiation by supporting the different bioenergetic requirements of highly proliferative neural stem cells compared to postmitotic neurons[3]. Mitochondrial dysfunction has been implicated in various aspects of neuronal and glial dysfunction, aging, as well as in the pathogenesis of neurodegenerative diseases[1], [2], [4]. However, how mitochondria cause and compensate for physiological and pathological challenges, and how this in turn affects neurogenesis, neural development, and nervous system function, remain poorly understood.

Multiple acyl-CoA dehydrogenase deficiency (MADD), also known as glutaric aciduria type II, is an autosomal-recessive inherited disorder caused by mutations in electron transfer flavoprotein (ETF) or electron transfer flavoprotein dehydrogenase (ETFDH)[5]. In mitochondria, ETF, located in the matrix, receives electrons from several dehydrogenases involved in fatty acid oxidation, choline and amino acid metabolism. ETF then transfers electrons to ETFDH, located in the inner mitochondrial membrane, and subsequently, electrons are passed to ubiquinone in the respiratory chain, leading to ATP production[6], [7]. As a result of ETF or ETFDH deficiency, the acyl-CoA dehydrogenases are unable to transfer the electrons generated by dehydrogenation reactions, resulting in the accumulation of various intramitochondrial acyl-CoA esters. There is secondary accumulation of free acids, and other conjugation products (acylcarnitine and acylglycine esters) in blood and urine, including large amounts of the lysine metabolic intermediate glutaric acid, giving the disease its name[5].

The clinical features of MADD are highly heterogeneous and have been classified as neonatal-onset form with (type I) or without (type II) congenital anomalies, and mild and/or late-onset form (type III). MADD consists of a large spectrum of symptoms, including hypotonia, hypoglycemia, cardiomyopathy, polycystic kidneys, and neurological manifestations such as symmetric warty dysplasia of the cerebral cortex, encephalopathy and leukodystrophy. While there have been case studies reporting the use of riboflavin[8] and sodium-3-hydroxybutyrate[9], [10] as treatment for MADD on a patient-by-patient basis, no systematic therapy has been validated. Moreover, despite the neurodevelopmental and cognitive dysfunction prominent in MADD patients, the anatomical, cellular and molecular abnormalities within the nervous system have not been well documented, and the mechanisms underlying neural phenotypes remain unexplored.

Here we report the genetic, cellular and molecular characterization of a zebrafish mutant *xav*. We had previously identified *xav* as a mutant that exhibits abnormal motility and aberrant neuromuscular synaptogenesis[11]. We found that the *xav* mutation resides in ETFDH, which is critical for fatty acid, amino acid and choline metabolism. Because dysfunction of this gene is responsible for human MADD, we performed several cellular and molecular analyses on *xav* mutants and fibroblast cells from MADD patients. Our results advance our understanding of how metabolism affects neural development, link mitochondrial dysfunction and the resulting increase in aerobic glycolysis to neurogenesis via the PPARG-ERK pathway, and suggest this pathway as a target for therapeutic intervention in human MADD.

## Materials and Methods

### Ethics Statement

All zebrafish husbandry and experimentation were conducted under a protocol approved by the University of Pennsylvania's Institutional Animal Care and Use Committee. Human fibroblasts were obtained from patients after written informed consent was obtained from parents/next of kin, under a protocol approved by the Institutional Review Board of Children's Hospital of Wisconsin and the Institutional Review Board of Children's Hospital of Philadelphia.

### Zebrafish Husbandry

Zebrafish were raised and maintained under standard conditions. The *xav* allele was previously described[11]. Wild type and mutant embryos were obtained from crosses between adult zebrafish.

### Fibroblasts from Human MADD Patients

Fibroblasts from a MADD patient 1, were obtained from Dr. William J. Rhead, Department of Pediatrics, Children's Hospital of Wisconsin. Fibroblasts from an age-matched control patient (an infant, <1 year of age, with no evident related disease) were obtained from Dr. Carsten Bonnemann, Children's Hospital of Philadelphia. Patient 1 is a deceased newborn with severe MADD, whose acylcarnitine profile showed elevations of C5- and C16- intermediates, and extremely low C2-carnitines (Dr. Rhead, personal communication). We found that patient 1showed mis-splicing of *ETFA* transcript, lacking the long isoform that contains exon2. Gene sequencing showed that patient 1 has a 52 C>T heterozygous mutation in exon2 that may cause the splicing defects. The same mutation has been reported in a MADD patient with neonatal neurological deterioration and metabolic acidosis[36]. Fibroblasts from passages 6 – 12 were grown to 80–100% confluency and used for metabolic or gene expression analyses as indicated. Fibroblasts were obtained from patients after written informed consent was obtained from parents/next of kin, under a protocol approved by the Institutional Review Board of Children's Hospital of Wisconsin and the Institutional Review Board of Children's Hospital of Philadelphia.

### Morpholino Injection

The morpholino antisense oligonucleotide (Gene Tools) targeting the *etfdh* intron2-exon3 junction (CTACCCCTGAAAACATTCAATTATA) was injected at the 1–2 cell stage at ∼8 ng.

### Plasma Acylcarnitine and Organic Acid Profiling

Sonicated fish (N = 40–80) were subjected to acylcarnitine and organic acid analysis. Acylcarnitines were analyzed by tandem mass spectrometry as butyl esters using the procedure initially developed for skin fibroblast acylcarnitine analysis[37]. Organic acids were analyzed as their trimethylsilyl derivatives by capillary gas chromatography- electron impact mass spectrometry using a procedure that was initially developed for urine and vitreous humour analysis[38].

### RNA Extraction and Quantitative RT-PCR (qRT-PCR)

RNA was extracted from a pool of 20 embryos with the RNeasy kit (Qiagen). The primers for qRT-PCR are shown in Supplemental Table S2. qRT-PCR was performed with the SuperScript® III Platinum® SYBR® Green One-Step qPCR Kit w/ROX (Invitrogen) and data was analyzed with 7500 Real-Time PCR System software (Applied Biosystems) using the 2^−ΔCT^ method, data were normalized to *β-actin1* for zebrafish and *ACTB* for fibroblasts.

### BrdU Labeling and Immunostaining

BrdU labeling was performed as described previously [39]. In brief, at ∼56–60 hpf, embryos were incubated with 10 mM BrdU in 10% DMSO in embryo medium for 30 min. on ice and then raised in embryo medium at 28.5°C for 30 min. The embryos were then fixed using 4% paraformaldehyde in PBS, pH = 7.4, followed by 2 hour incubation in 2 M HCl. Embryos were anesthetized, fixed and immunostained as described previously[11] using antibodies against BrdU (mouse monoclonal Developmental Studies Hybridoma Bank (DSHB); rabbit polyclonal, Abcam) and dp-ERK (Sigma) and the appropriate fluorescently conjugated secondary antibody (Jackson Labs). Unless otherwise stated, each figure panel showing immunostaining is a single plane projection of a confocal z-stack of 20–60 1 µm thick planes (Leica TCS 4D system) and was assessed using interactive software (Metamorph).

### Western Blotting

To prepare protein, embryos were triturated in lysis buffer (100 mM pH 8 Tris, 1% SDS, 10 mM EDTA, 50 mM DTT). Protein quantity was assessed (D_c_ Protein Assay; Bio-Rad) and proteins were separated by SDS-PAGE (4–10% gradient gel), transferred to a nitrocellulose membrane and probed with antibodies against AMPKα (Cell Signaling), phospho-AMPKα (Thr172) (Cell Signaling), PPARG (Santa Cruz), dp-ERK (Sigma), phospho-STAT3 (Tyr705) (MBL) and/or actin (Sigma). After washing, blots were incubated in AP-conjugated secondary antibody (Jackson Labs), and then visualized using chemiluminescence (Western*Star* detection system; Applied Biosystems).

### ROS Labeling

Embryos were incubated in embryo medium containing 10 µM of dyhydrorhodamine123 (DHR123, Invitrogen) for 2 hrs at 28°C, then washed with embryo medium several times and examined with confocal microscopy.

### PPARG Pharmacology

Embryos were incubated in 25 µ m PPARG antagonist BADGE[28] or agonist 10 µM Ciglitizone[40] at ∼24 hpf until the desired developmental stage.

### Statistical Analyses

All statistical analyses were performed with Prism software (Microcal) and Excel (Microsoft).

## Results

### External Phenotypes, Genetic Cloning and Morpholino Phenocopy of *xav*

**Figure 1:**
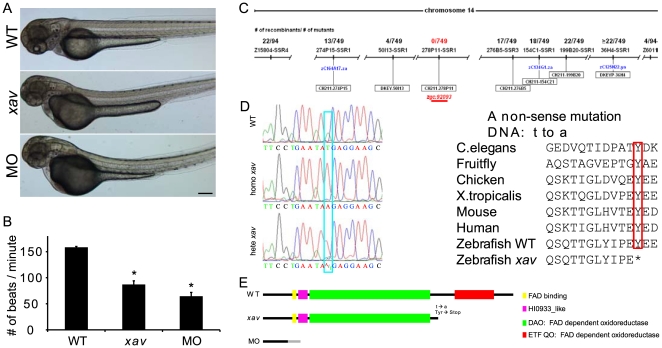
External phenotype, genotype, cloning and morpholino phenocopy of *xav* mutants. **A.** External *xav* and *etfdh* morphant (MO) phenotypes at ∼60 hpf include a bent and thinner tail and smaller head and eyes. Scale bar = 100 µm. **B.** *xav* mutants and MO exhibit slower heart beat (WT 158±6 beats/minute, N = 13 embryos; *xav* 87±7; N = 14 embryos; MO 65±7, N = 14 embryos; one-way ANOVA, followed by Dunn's pairwise comparison, * p<0.001. **C.** Genetic and physical map of the *xav* (zgc∶92093) locus (red), including microsatellite and SSR markers, number of recombinants, and BAC clones from the T51 radiation hybrid panel. **D.** *etfdh* mutation in *xav* is a T to A mutation (blue box) resulting in a premature stop codon (red box). The amino acid sequence of etfdh is highly conserved among several species, from C. elegans to human. **E.** Schematic location of *xav* mutation, resulting in truncation of the C terminal. *etfdh* MO is predicted to give rise to a protein fragment lacking all functional domains.

We previously identified *xav* in a small scale genetic screen due to its abnormal swimming behavior and decreased number of neuromuscular synapses[11]. Externally, *xav* mutants cannot be distinguished from WT until after 48 hpf, when they start to exhibit a bent tail and slower heart beat, phenotypes that become progressively more severe (Figure 1A, 1B).

Positional cloning strategies (Figure S1; Sup. Table S1) identified *xav* mutation resides in *zgc∶92093*, which encodes electron-transfer-flavoprotein dehydrogenase (etfdh) (Figure 1C). There is a T to A transversion at position 1305 which introduces a premature stop codon (Y435X) in the 617 amino acid protein (Figure 1D, 1E).

Besides the truncation of the protein caused by the *xav etfdh* mutation, we also found that the overall abundance of *etfdh* mRNA in *xav* is dramatically reduced. Real-time quantitative RT-PCR (qRT-PCR) showed that there is 80% reduction of *etfdh* mRNA (Figure S2A), likely due to nonsense mediated decay[12]. Furthermore, *xav* mutants showed nonsense mediated alternative splicing. As a result of the mutation, which resides in exon11, the exon10-exon13 junctions are mis-spliced in mutants, resulting in transcripts that are predicted to encode proteins lacking critical domains or truncated (Figure S2B). These results suggest that *xav* contains a likely loss of function mutation in *etfdh*.

To confirm whether *etfdh* is the gene mutated in *xav*, we designed a splice-blocking morpholino against *etfdh* and compared the phenotypes in morphants and mutants. Injection of 8 ng *etfdh* MOI2E3 in WT embryos, which targets intron2-exon 3 junction, results in >80% reduction of the normal transcript at 2 and 3 dpf, producing a mis-spliced transcript that lacks exon3 (Figure S3C, 3D), resulting in predicted protein fragment lacking all functional domains (Figure 1E). *etfdh* morphants not only showed bent tail and reduced heart beat (Figure 1A, 1B), but also exhibited aberrant swimming behavior and reduced neuromuscular synaptogenesis (see Supplemental Results Text S1; Figure S6; and data not shown), as in *xav* mutants (Sup. Video S1; Sup. Video S2). These results indicate that *etfdh* mutation is responsible for *xav* phenotypes.

### *xav* Mutants Exhibit Plasma Acylcarnitine and Organic Acid Profiles Similar to Those in MADD Patients

Given the fact that *xav* and MADD patients have mutation in the same gene, we asked whether *xav* mutants exhibit phenotypes similar to those seen in human patients. Clinically, MADD is diagnosed by the plasma acylcarnitine profile, the urine organic acid profile and acylglycine analysis. These analyses were thus performed in *xav* mutants compared to WT embryos.

**Figure 2:**
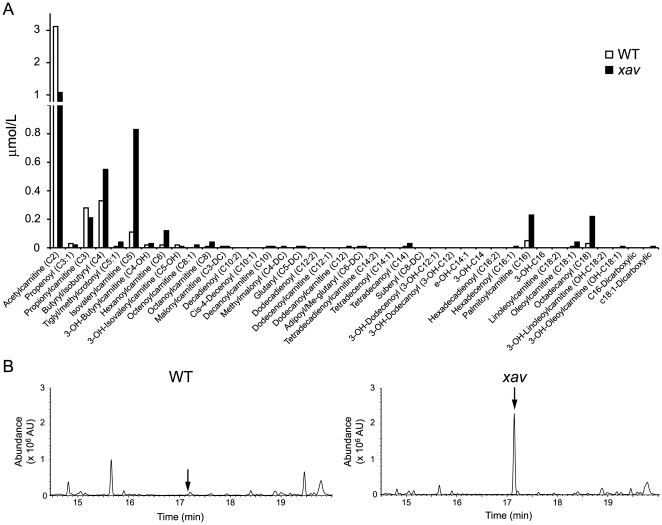
*Xav* mutants display abnormal acylcarnitine and organic acid profile. **A.** Representative acylcarnitine profile from homogenates of WT and *xav* mutant embryos using tandem mass spectrometry, showing a markedly higher level of several intermediate acyl-fatty acid species in *xav* mutants including C4, C5, C6, C8, C14, C16 and C18, and a reduction of C2 acetylcarnitine (pool of ∼100 embryos for *xav* mutant and WT at ∼56 hpf). **B.** Representative organic acid profile from homogenates of WT and *xav* mutant embryos using gas chromatography electron impact mass spectrometric analysis, showing an elevation of the level of glutaric acid in *xav* mutants (black arrow) (pool of ∼100 embryos for *xav* mutant and WT at ∼56 hpf).

Tandem mass spectroscopy of plasma acylcarnitine detected a markedly higher level of a spectrum of intermediate acyl-fatty acid species in *xav* mutants, including C4, C5, C6, C8, C14, C16 and C18, together with a drastic reduction of C2 acylcarnitine (Figure 2A), suggesting dysregulation of mitochondrial β-oxidation and alterations in multiple intermediary mitochondrial metabolic pathways in *xav*, similar to that observed in MADD patients[5].

Gas chromatographic analysis of organic acids from embryo homogenates detected a dramatic elevation of glutaric acid in *xav* (Figure 2B). Further quantification showed the glutaric acid content in *xav* is 0.99 µg/embryo, but <0.05 µg/embryo in WT, resembling the glutaric acidemia seen in MADD patients[5].

While acylglycine analysis showed elevated acylglycine levels in MADD patients, no acylglycine was detected in either WT or *xav* embryos. This suggests that the function of glycine-N-acyltransferase, which converts acyl-CoA and glycine to CoA and acylglycine, may not be conserved between zebrafish and humans. Moreover, kidney defects were observed at the organ structural and cellular level in *xav* mutants compared to WT embryos at ∼60 hpf (Figure S3), consistent with a polycystic kidney phenotype that is prominent in MADD patients. Together, these analyses show that the *etfdh* mutation in *xav* results in MADD like metabolic and kidney defects.

### *xav* Mutants Exhibit Several Hallmarks of Mitochondrial Dysfunction

**Figure 3:**
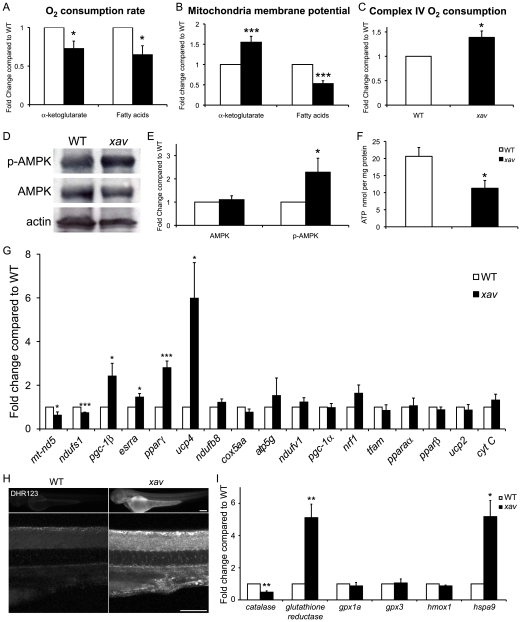
*xav* mutants exhibit mitochondrial dysfunction. **A.** Measured rates of state 3 respiration supported by oxidation of α-ketoglutarate and fatty acids (palmitoylcarnitine and octoylcarnitine (each + malate)) showed a 30–35% reduction in *xav* mutant compared to WT embryos at ∼56 hpf (values normalized to WT; α-ketoglutarate in *xav*, 0.72±0.10; N = 4 experiments 50–100 embryos each; Student's t test, * p = 0.03; fatty acid 0.65±0.10 in *xav*; N = 4 experiments 50–100 embryos each; Student's t test, * p = 0.02). **B.** Membrane potential generated by oxidation of α-ketoglutarate and fatty acids was estimated using TMRE and spectrofluorometry, showing a 55% increase in *xav* compared to WT at ∼56 hpf (values normalized to WT; α-ketoglutarate *xav* 1.55 ± 0.15; N = 4 experiments 50–100 embryos each; Student's t test, *** p<0.001) and a 50% reduction (fatty acids *xav* 0.53±0.07; N = 5 experiments 50–100 embryos each; Student's t test, *** p<0.001). **C.** Measured O_2_ consumption rates with ascorbate/TMPD showed a 40% increase in *xav* compared to WT at ∼56 hpf (values normalized to WT; *xav* 1.39±0.13; N = 6 experiments 50–100 embryos each; Student's t test, * p = 0.014). **D–E.** Western blot showed that while the total level of AMPK is unchanged in *xav*, the amount of activated AMPK (phospho-AMPKα^Thr172^) protein is significantly increased. Quantification showed a 2.3 fold increase in *xav* mutants compared to WT embryos (values normalized to WT; *xav* 2.3±0.6; N = 3 experiments 30 embryos each; Student's t test, * p<0.05). **F.** Levels of ATP production showed a ∼45% reduction in *xav* mutants compared to WT embryos (WT 20.6±2.6 nmol per mg protein; *xav* 11.3±2.3 nmol per mg protein; N = 9 replicates, 50–100 embryos each, Student's t test, * p<0.05). **G.** mRNA levels of genes involved in mitochondrial function and biogenesis were analyzed with qRT-PCR. A ∼30–40% reduction was observed in *mt-nd5* and *ndufs1*, and a ∼2.4, 1.5 and 2.8 fold increase was observed in *pgc-1β*, *esrrα* and *pparγ* in *xav* mutants compared to WT. Expression of zebrafish *uncoupling protein 4* (*fucp4*) was increased ∼6 fold, while expression of *ucp2* was unchanged (N = 3–4 replicates, 20 embryos each; Student's t test, * p<0.05, ** p<0.01, *** p<0.001). **H.** The oxidative fluorescent dye DHR-123 was used to measure cellular superoxide production in live embryos and showed higher cellular superoxide levels in *xav* mutants compared to WT, especially in the nervous system, including in the spinal cord. Scale bar = 100 µm. **I.** Expression profile of genes known to be involved in the ROS pathway was assayed with qRT-PCR and showed a ∼50% reduction of *catalase* transcripts and ∼5 fold increase of *glutathione reductase* and *hspa9* in *xav* compared to WT (N = 3 replicates, 20 embryos each; Student's t test, * p<0.05, ** p<0.01).

Given that *etfdh* functions in mitochondria, and is involved in electron transport from fatty acid and amino acids, we asked whether mitochondrial function is abnormal in *xav* mutants. First, we evaluated the respiratory and phosphorylation activities in homogenates of *xav* mutants and WT embryos at ∼56 hpf, by measuring O_2_ consumption in the presence of α-ketoglutarate or fatty acids (palmitoylcarnitine and octoylcarnitine) as substrates (see Supplemental Experimental Procedures Text S1). ADP-stimulated (state 3) rates of O_2_ consumption were decreased ∼30–35% in *xav* mitochondria with each of these substrates (Figure 3A; Figure S4A). The blunted rates of state 3 respiration in *xav* mitochondria (Figure S4A) suggest that oxidative phosphorylation is compromised in *xav* mutants.

Mitochondria establish a transmembrane potential, which is generated when the energy released by the flow of electrons through the electron transport chain is used to pump protons out of the mitochondrial inner membrane through complexes I, III, and IV, and is utilized to support critical cellular processes, such as the production of ATP and Ca^2+^ uptake. We estimated mitochondrial membrane potential using the positively charged fluorescent probe tetramethylrhodamine ethyl ester (TMRE) in *xav* mutant and WT embryos at ∼56 hpf. Mitochondrial transmembrane was estimated as the difference in fluorescence signals following substrate energization and complete depolarization and collapse of the membrane potential following addition of uncoupler. We found a ∼50% reduction of membrane potential generated by oxidation of fatty acid (palmitoylcarnitine and octoylcarnitine) (Figure 3B), consistent with the observed decreases in state 3 O_2_ consumption with these substrates. Surprisingly, however, despite the decrease in the rate of state 3 O_2_ consumption observed with α-ketoglutarate in *xav*, mitochondrial membrane potential was estimated to be increased by ∼50% (Figure 3B). This result suggests that the transmembrane potential generated when α-ketoglutarate is oxidized by the electron transport chain may not be properly utilized for ADP phosphorylation in *xav* mutants.

Despite reduced rates of state 3 O_2_ consumption observed following the oxidation of α-ketoglutarate and fatty acid in *xav*, the activity of complex IV was increased by 40% when assayed directly after addition of ascorbate/N,N,N′,N′-tetramethyl-p -phenylenediamine (TMPD) (Figure 3C), suggesting a possible compensatory metabolic response to augment respiratory capacity in *xav* mutants. Consistent with this idea, at ∼56 hpf, a higher level of F1-F0 ATPase (complex V) protein was detected by immunostaining in *xav* mutants compared to WT (Figure S4B). This likely reflects an adaptation to insufficient respiration, and is consistent with compensation.

To further assess mitochondrial defects, we examined the activity of AMP-activated protein kinase (AMPK), which is an evolutionarily conserved metabolic sensor that responds to alterations in cellular energy levels to maintain energy balance. AMPK activation occurs in response to ATP depletion and a concomitant increase in the AMP/ATP ratio. Once activated, AMPK phosphorylates numerous downstream substrates thereby initiating a series of responses aimed at restoring cellular energy balance by switching off ATP-consuming, anabolic pathways, such as fatty acid synthesis and protein synthesis and switching on ATP-generating pathways such as fatty acid oxidation and glycolysis. Biochemical analyses showed that while the total level of AMPK is unchanged in *xav*, the level of activated AMPK (phospho-AMPKα^Thr172^) protein is significantly increased (Figure 3D, 3E). This result suggests that there is a homeostatic regulation in *xav* mutants, in response to alterations in metabolism. In addition, measurement of ATP levels showed a ∼45% reduction in *xav* mutant compared to WT embryos (Figure 3F), despite a compensatory increase of complex IV, complex V and activated, p-AMPK protein levels. Together, these results suggest that the *etfdh* mutation in *xav* results in metabolic reprogramming, which is not restricted to and cannot fully compensate for the defect in the fatty acid oxidation.

In order to gain more insight into the mitochondrial dysfunction in *xav* and the underlying molecular mechanisms, we profiled the expression of genes known to be involved in mitochondrial function and biogenesis with qRT-PCR (Figure 3G). We found a ∼30–40% reduction of *mt-nd5* and *ndufs1*, two mitochondria encoded genes that belong to complex I. Mutations of these genes are associated with complex I deficiency and mitochondrial encephalomyopathy, lactic acidosis, and stroke-like episodes (MELAS), two disorders which have neurological manifestations[13], [14], [15]. The expression of *pgc-1β*, *esrrα* and *pparγ*, genes involved in transcriptional regulation of energy metabolism, were increased ∼2.4, 1.5 and 2.8 fold, respectively. Interestingly, expression of zebrafish *uncoupling protein 4* (*fucp4*), which is proposed to be responsible for uncoupling of respiration from ATP synthesis and thus protect against reactive oxygen species (ROS) production, showed a ∼6 fold increase, while *ucp2* expression remained unchanged. The closest homologue of *fucp4* in humans, *UCP3*, has been reported to be upregulated in MADD patients[16]. These results document mitochondrial dysfunction at the level of alteration of gene expression in *xav* mutants as a result of *etfdh* mutation.

Impairment of mitochondrial metabolism, including β-oxidation, may result in greater oxidative stress[17], which increases generation of ROS. The oxidative fluorescent dye dihydrorodamine-123 (DHR-123) was used to qualitatively address cellular superoxide production. DHR-123 labeling in live embryos showed higher cellular superoxide levels in *xav* mutants compared to WT, especially in the nervous system, including in the spinal cord (Figure 3H). We next assessed the expression profile of genes known to be involved in the ROS pathway. qRT-PCR analyses showed a 50% reduction of *catalase* transcripts and 5-fold increase of *glutathione reductase* (Figure 3I), both of which are known to function in ROS scavenging. We also found a 5 fold increase of *hspa9* (Figure 3I), mutation of which produces an increase in ROS in blood cells[18]. These results further confirm that *etfdh* mutation results in mitochondrial dysfunction and subsequent oxidative stress in *xav* mutants. Moreover, the increased membrane potential observed with α-ketoglutarate + malate in *xav*, as discussed above, may also contribute to the increased ROS production. Mitochondrial ROS formation is favored by a high transmembrane potential: increased membrane potential decreases electron flow and decreased electron flow in turn increases the half-life of partially reduced components of the electron transport chain, thereby increasing the probability these carriers may donate an electron to O_2_ to form superoxide[19], [20].

### Human MADD Fibroblast Cells Display Similar Mitochondrial Dysfunction as *xav* Mutants

We next asked whether the mitochondrial abnormalities observed in *xav* are similar to those in MADD patients. As analyses of MADD patient tissues are rare, and postmortem tissues are not available, we performed analyses on fibroblast cells from Patient 1, and a control patient.

**Figure 4:**
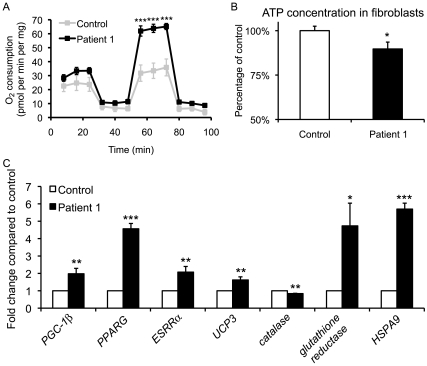
Human MADD fibroblast cells display similar mitochondrial defects as *xav* mutants. **A.** Oxygen consumption was measured in intact control and MADD Patient 1 fibroblasts under basal conditions, following the addition of the mitochondrial ATP synthase inhibitor oligomycin (0.5 µg/ml), in the presence of the uncoupler CICCP (3 µM) to maximally stimulate respiration and following the addition of complex I inhibitor rotenone (100 nM) to assess residual non-mitochondrial oxygen consumption. A 2-fold increase was observed in Patient 1 fibroblasts compared to control, after uncoupler CICCP treatment, which measures the maximal uncontrolled mitochondrial respiratory capacity (N = 3 experiments, 7–9 replicates of cells from passage 7–12; Student's t test, *** p<0.001). **B.** Measurement of ATP levels showed a ca. 10% reduction in MADD Patient 1 fibroblast cells compared to control (N = 14 replicates of cells from passage 7–12; Student's t test, * p<0.05). **C.** Gene expression assayed by qRT-PCR revealed that *PGC-1β*, *PPARG*, *ESRRα*, *UCP3* were increased ∼2, 4.6, 2 and 1.6 fold in Patient 1 compared to control. The expression of ROS related genes are also altered in Patient 1, with a ca. 20% decrease in *catalase* and 4.7 and 5.7 fold increase in *glutathione reductase* and *HSPA9* expression (N = 3–7 replicates of cells from passage 7–12; Student's t test, * p<0.05, ** p<0.01, *** p<0.001).

Fibroblasts from Patient 1 exhibited markedly increased uncontrolled mitochondrial respiration as elicited by the uncoupler carbonyl cyanide *m*-chlorophenylhydrazone (CICCP) compared to control fibroblasts (Figure 4A), resembling the increased complex IV activity and complex V expression observed in *xav*. The observation that uncoupler-stimulated respiration in fibroblasts from Patient 1 was so significantly above the basal respiratory rate suggests that proton translocation through the mitochondrial ATP synthase *in situ* limits state 3 oxygen consumption, and therefore ATP synthesis, in these cells. The addition of uncoupler relieves/removes any constraint on respiration which may be imposed by proton movement through the ATP synthase, thereby increasing respiration to a maximal level. Basal ATP levels were slightly but significantly reduced in Patient 1 fibroblasts (Figure 4B). These results suggest altered respiratory capacity and compensation in MADD patients as in *xav* mutants. Moreover, while electron transport chain is compromised, cells *in vitro* are more readily able to maintain ATP homeostasis than *in vivo*, either by upregulating reserve respiratory capacity, by shifting to glycolysis (as discussed in the following section) or by reducing dependence on fatty acids.

Gene expression profiling revealed that *PGC-1β*, *PPARG*, *ESRRα*, *UCP3* were all significantly increased in Patient 1 (Figure 4C), as in *xav*. Furthermore, the expression of ROS related genes are also altered in Patient 1, as in *xav*, in particular decreased *catalase* and increased *glutathione reductase* and *HSPA9* (Figure 4C). These results extend our understanding of the extent of mitochondrial dysfunction in human MADD, and show further that this dysfunction is similar at the metabolic and gene expression level in *xav* and MADD patients. Furthermore, this indicates that the dysfunction is independent of the particular gene resulting in human MADD, as both ETF and ETFDH defects demonstrate similar abnormalities in these studies.

### MADD Fibroblasts and *xav* Mutants Exhibit Increased Aerobic Glycolysis

**Figure 5:**
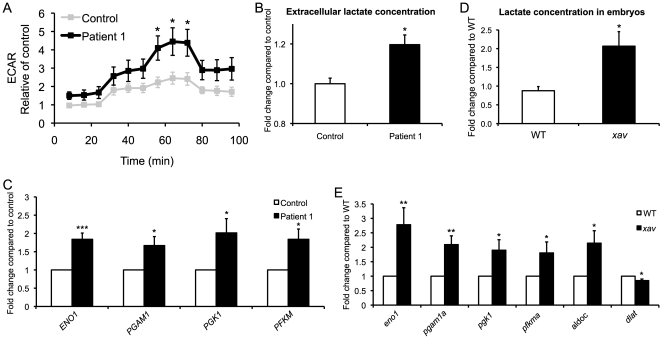
Increased aerobic glycolysis in MADD fibroblasts and *xav* mutants. **A.** ECAR was measured in intact control and MADD Patient 1 fibroblasts under basal conditions, following the addition of the mitochondrial inhibitor oligomycin (0.5 µg/ml), in the presence of the uncoupler CICCP (3 µM) and following the addition of the complex I inhibitor rotenone (100 nM). A trend towards higher ECAR rate was seen under basal conditions, and a significant 2-fold increase was observed after uncoupler CICCP treatment in Patient 1 fibroblasts compared to control. (N = 3 experiments, 7–9 replicates of cells from passage 7–12; Student's t test, * p<0.05). **B.** Lactate present in the culture medium showed a ∼20% increase in the basal lactate production in Patient 1 fibroblasts compared to control (N = 2 experiments, 5–6 replicates of cells from passage 7–8; Student's t test, * p<0.05). **C.** qRT-PCR analyses of genes involved in glycolysis showed glycolytic enzymes *enolase 1* (*ENO1*), *phosphoglycerate mutase 1* (*PGAM1*), *phosphoglycerate kinase 1* (*PGK1*) and *phosphofructokinase* (*PFKM*), were increased by ∼1.9, 2.7, 3.4 and 3.1 fold respectively in Patient 1 fibroblasts compared to control (N = 5 replicates of cells from passage 7–9; Student's t test, * p<0.05, *** p<0.001). **D.** Lactate levels showed a ∼2.4 fold increase in *xav* mutants compared to WT embryos at ∼56 hpf (N = 7–9 replicates, 30–100 embryos each; Student's t test, * p<0.05). **E.** qRT-PCR analyses of gene expression revealed increased expression of glycolytic enzymes *eno1*, *pgam1a*, *pgk1*, *pfkma*, and *fructose-biphosphate aldolase C* (*aldoc*) by 2.8, 2, 1.9, 1.8 and 2.1 fold respectively, as well a ∼20% decrease in *dihydrolipoamide S-acetyltransferase* (*dlat*), that belongs to the pyruvate dehydrogenase complex, which links glycolysis to tricarboxylic acid (TCA) cycle, in *xav* mutants compared to WT embryos at ∼56 hpf (N = 3–7 replicates, 20 embryos each; Student's t test, * p<0.05, ** p<0.01).

During the course of measuring mitochondrial oxidative responses, we noticed that there was also a significant increase in the extracellular acidification rate (ECAR) in Patient 1 fibroblasts, especially in the presence of an uncoupler (Figure 5A). ECAR reflects changes in proton concentration and is used as readout of lactate production as this dominates the acidification and is thus used as a surrogate for glycolysis[21]. While the increase in ECAR in Patient 1 fibroblasts suggested that aerobic glycolysis is increased, we performed two additional analyses to further examine this possibility. First, the amount of lactate secreted into the culture medium was directly measured in Patient 1 fibroblasts. Basal lactate production was increased by ∼25%, consistent with increased glycolysis (Figure 5B). Second, the expression profile of genes critical for glycolysis or in the glycolytic pathway was assessed using qRT-PCR. mRNA for glycolytic enzymes *enolase 1* (*ENO1*), *phosphoglycerate mutase 1* (*PGAM1*), *phosphoglycerate kinase 1* (*PGK1*) and *phosphofructokinase* (*PFKM*) were all significantly increased in MADD patients (Figure 5C). These results suggest that aerobic glycolysis is increased in fibroblasts from MADD patients.

To determine whether glycolysis is also elevated in *xav* mutants, the amount of lactate was directly measured and was found to be increased by ∼2.4 fold in *xav* mutants compared to WT embryos at ∼56 hpf (Figure 5D). Gene expression analyses by qRT-PCR revealed significant changes in expression of several glycolytic genes. mRNA for the glycolytic enzymes *eno1*, *pgam1a*, *pgk1* and *pfkma*, as well as *fructose-biphosphate aldolase C* (*aldoc*) was significantly increased (Figure 5E). mRNA for *dihydrolipoamide S-acetyltransferase* (*dlat*), that belongs to the pyruvate dehydrogenase complex (PDH) which links glycolysis to tricarboxylic acid (TCA) cycle, was significantly decreased (Figure 5E). Together, these data suggest that in both human MADD cells and *xav* mutants, the defect in fatty acid oxidation compromises oxidative phosphorylation, leading to an upregulation in aerobic glycolysis as an alternative energy source, possibly as a consequence of AMPK activation, in an attempt to compensate for this metabolic insuffiency. Furthermore, the decreased expression in of *dlat* in *xav* will likely result in decreased activity of PDH, in turn diverting pyruvate away from mitochondria, decreasing flux through the Krebs cycle and thereby decreasing delivery of reducing equivalents in the form of NADH to the electron transport chain. Shunting of pyruvate away from mitochondria would thus contribute to the conversion of glucose to lactate and further exacerbate any underlying defects in the electron transport chain by restricting oxidation of a very important mitochondrial substrate, favoring a glycolytic phenotype.

### *xav* Mutants Exhibit Increased Neural Proliferation

Many proliferating cells, including some cancer cells, utilize aerobic glycolysis, which while an inefficient way to generate ATP compared to oxidative phosphorylation, has been suggested to have the advantage of generating a number of biosynthetic intermediates which can be used to incorporate nutrients into the cell biomass, a phenomenon known as the Warburg effect[22]. Because a shift away from oxidative phosphorylation to an increased dependence upon aerobic glycolysis may affect proliferation, and because of the striking neural phenotypes observed in *xav* mutants, including reduced neuropil staining, abnormal glial patterning, reduced motor axon branching and neuromuscular synaptogenesis, increased cell death and progressive paralysis (Supplemental Results Text S1 and Figure S5, S6, S7, S8, S9), we asked whether neural cell proliferation is increased in *xav* mutants compared to WT embryos using BrdU incorporation.

**Figure 6:**
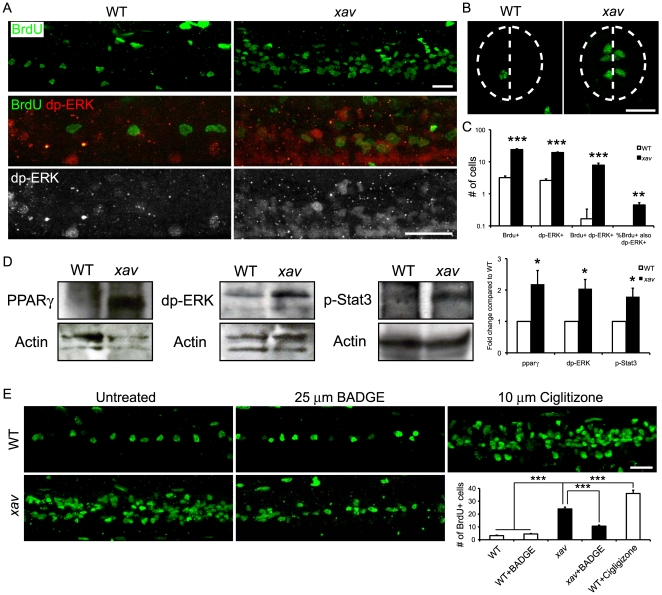
*xav* mutants exhibit increased neural cell proliferation as a result of increased glycolysis, due to perturbation of the PPARG-ERK pathway. **A.** Whole mount embryos labeled with BrdU to mark cells undergoing proliferation. The number of BrdU+ cells was significantly increased in the nervous system, especially in the spinal cord, in *xav* mutants compared to WT embryos at ∼56–60 hpf. Increased dp-ERK+ cells and BrdU+/dp-ERK+ double labeled cells in the spinal cord in *xav* mutants compared to WT embryos at ∼56–60 hpf. **B.** In spinal cord cross-sections from *xav* mutants and WT embryos at ∼56–60 hpf, BrdU+ cells are distributed peri-ventricularly, suggesting that they are likely to be neural progenitor cells. Dashed line outlines the spinal cord and indicates the midline. Scale bar = 20 µm. **C.** Quantification of BrdU+ and dp-ERK+ cells at ∼56–60 hpf. Per spinal cord hemisegment: BrdU+ cells WT 3.2±0.4, *xav* 24±1.4; dp-ERK+ cells WT 2.6±0.3, *xav* 19.4±0.8; BrdU+/dp-ERK+ WT 0.2±0.2, *xav* 7.8±1.2. Percent of BrdU+ cells that are also dp-ERK+: WT 3.6%±3.6%, *xav* 45%±8% (N = 4-19 embryos, >2 carrier pairs; Student's t test, *** p<0.001). **D.** Western blot analyses of pparγ, dp-ERK and phospho-STAT3 expression showed dramatic increase in *xav* compared to WT embryos at ∼56–60 hpf (N = 3 replicates, 30 embryos each; Student's t test, * p<0.05). **E.** BrdU labeling of proliferating cells in whole mounts of spinal cord of WT embryos, *xav* mutants, WT treated with 25 µm BADGE, *xav* mutants treated with 25 µm BADGE and WT treated with 10 µm Ciglitizone at ∼56–60 hpf. Embryos were raised in BADGE or Ciglitizone from 24 to 60 hpf. Per spinal hemisegment: BrdU+ cells WT 3.2±0.4, WT + BADGE 4.5±0.3, *xav* 24±1.4, *xav *+ BAGDE 10.6±0.9, WT + Ciglitizone 36±2.5 (N = 6–19 embryos, >3 carrier pairs; one-way ANOVA, followed by Bonferroni's multiple comparison test, *** p<0.001).

The number of BrdU+ cells was significantly increased throughout the nervous system, in particular in the spinal cord, of *xav* mutants compared to WT embryos at 56–60 hpf (Figure 6A, 6B). While in ∼56–60 hpf WT embryos, there are ∼3 BrdU+ cells in the spinal cord per hemisegment, there are ∼24 in *xav* mutant embryos at the same developmental stage, resulting in an expansion of the proliferating cellular domain dorso-ventrally and rostro-caudally (Figure 6A). The peri-ventricular location of these BrdU+ cells suggests that they are likely to be neural progenitor cells[23], [24]. These observations suggest that neural cell proliferation is increased in the nervous system of *xav* mutants.

### Relationship between Increased Activation of the PPAR-ERK Pathway and Increased Neural Proliferation in *xav* Mutants

In order to explore the mechanistic relationship between metabolic dysfunction and increased neural proliferation in *xav*, we focused on the PPARG-ERK pathway. PPARG is a known regulator of the cell cycle and apoptosis, and is highly expressed in many cells, including neurons and some human cancer cells[25]. High levels of PPARG expression have been reported in embryonic mouse brain and neural progenitors, while very low levels have been reported in adult mouse brain[26], [27]. Recently, PPARG has been shown to regulate neural proliferation *in vitro*, through the activation of ERK and STAT3[28]. Because our qRT-PCR analyses showed a dramatic increase in *PPARG* expression in *xav* mutant embryos and MADD patient fibroblasts, this pathway was further examined using Western blot and immunostaining analyses.

Western blot analyses showed that PPARG protein levels were elevated in *xav*, consistent with elevated mRNA expression (Figure 6D). Western blot and immunostaining analyses were also used to assess ERK activation using a phospho-ERK specific antibody (dp-ERK)[29]. The amount of dp-ERK protein was significantly increased, and dp-ERK immunoreactivity was increased in the spinal cord, of *xav* compared to WT embryos at ∼56–60 hpf (Figure 6A, 6D). BrdU labeling of dividing cells and dp-ERK immunostaining in *xav* mutant embryos at ∼56–60 hpf revealed a significant increase in the number of double positive BrdU+/dp-ERK+ cells, from ∼0 in WT to ∼8 per hemisegment in *xav* mutant embryos at ∼56–60 hpf (Figure 6A, 6C). Furthermore, 45% of the BrdU+ cells were dp-ERK positive. Western blot analyses were then used to assess the level of activated STAT3 protein, using an antibody against phosphorylated STAT3 (phospho-STAT3^Tyr705^)[30]. The amount of phosphorylated STAT3 protein was significantly increased in *xav* embryos (Figure 6D). These results suggest that the PPARG-ERK pathway is dysfunctional in *xav* mutants, and is associated with increased cell proliferation.

PPARG antagonists and agonists were used to assess the mechanistic relationship between the PPARG-ERK pathway and the increased cell proliferation observed in *xav* mutant embryos. We found that 25 µM of the PPARG antagonist 2,2-Bis[4-(glycidyloxy)phenyl]propane, 4,4′-isopropylidenediphenol diglycidyl ether (BADGE), when applied from 24–60 hpf, significantly reduced the number of BrdU+cells in the spinal cord of *xav* mutants, from ∼24 to ∼10 cells per hemisegment (Figure 6E). Furthermore, 10 µM of the PPARG agonist Ciglitizone, when applied to WT embryos from 24–60 hpf, significantly increased cell proliferation in the spinal cord, from ∼3 to 36 cells per hemisegment (Figure 6E). Moreover, we found that BADGE, when applied from 24–60 hpf, significantly reduced the proportion of *xav* mutants that were paralyzed at ∼60 hpf, from 18% to 4%, and also delayed the onset of paralysis by ∼12 hours (Figure 6F; see also Supplemental Results Text S1).

Together, these data suggest that aberrant activation of the PPARG-ERK pathway underlies, at least in part, the cell proliferation and behavioral defects that are prominent in *xav* mutants, linking metabolic and mitochondrial dysfunction with defects in nervous system development, and possibly other organ system development, in *xav* mutants and MADD patients.

## Discussion

We report that the *xav* mutation causes a loss of ETFDH function and defective electron transfer, and that both *xav* and fibroblasts from a phenotypically severe MADD patient have similar metabolic defects and mitochondrial dysfunction, including altered energy metabolism, dysregulated ROS production and altered expression of genes critical for mitochondrial function. *xav* mutants and MADD fibroblasts exhibit increased aerobic glycolysis, similar to the Warburg effect observed in cancer cells, leading to excessive neural proliferation in *xav*, mediated by upregulation of the PPARG-ERK pathway. *xav* mutants also display motility defects culminating in paralysis, abnormal glial patterning, reduced motor axon branching and neuromuscular synapse number, but muscle fiber and neuromuscular synapse function appear normal. While there is increased apoptosis throughout the nervous system, many of these phenotypes are independent of cell death, as they are not rescued when cell death is blocked. Strikingly, a PPARG antagonist attenuates aberrant neural proliferation and alleviates paralysis in *xav*, while PPARG agonists increase neural proliferation in wild type embryos. This work provides further insights into the relationship between metabolism and neural development, specifically that mitochondrial dysfunction leads to an increase in aerobic glycolysis which affects neurogenesis, at least in part through the PPARG-ERK pathway.

While it is not surprising that mutations in ETF genes or ETFDH would lead to impaired fatty acid, choline and amino acid metabolism, it is interesting that a broader metabolic defect is also present. Our finding that mitochondrial oxidative phosphorylation stimulated by substrates other than fatty acids is also compromised, and that there is a compensatory elevation of complex IV activity, complex V expression and glycolysis further supports the idea that there is crosstalk between bioenergetic and metabolic pathways[31].

In most cancer cells and other rapidly proliferating cell populations, ATP is produced primarily by aerobic glycolysis followed by lactate fermentation, rather than by mitochondrial oxidative phosphorylation as in normal, differentiated cells, a phenomenon known as the Warburg effect. The Warburg effect has been proposed as an adaptive strategy to facilitate the uptake and incorporation of essential biosynthetic intermediates needed for increasing cell biomass and proliferation[22]. Despite understanding that proliferating cells switch from oxidative phosphorylation to aerobic glycolysis, the underlying triggers, effectors and mediators of this switch remain elusive. We show using several cellular and molecular assays that *xav* mutants and MADD fibroblasts exhibit a similar switch characterized by enhanced aerobic glycolysis accompanied by reduced mitochondrial oxygen consumption, the consequence of which is increased cell proliferation, in particular in the nervous system of *xav* mutants.

While the molecular basis of the metabolic pathology in MADD can be explained by the malfunction of fatty acid, choline and amino acid metabolism as a result of *ETF* or *ETFDH* mutation [5], it remains unclear why individuals with MADD exhibit other defects, especially neurological defects including cortex dysplasia, encephalopathy and leukodystrophy[32], [33], [34], [35]. In *xav* mutants, the deficiency of fatty acid metabolism and oxidative phosphorylation may force cells to augment glycolysis as an alternative energy source for energy and biosynthetic intermediates to preserve viability. This switch may alter the balance between cell proliferation and differentiation, a major regulator of which is PPARG signaling. In both *xav* mutants and MADD fibroblasts, PPARG expression is increased at the mRNA and protein level, and activation of downstream effectors such as ERK and STAT3 are also increased. We showed that PPARG elevation underlies, in large part, the increase in cell proliferation in the nervous system of *xav* mutants by antagonizing this pathway in *xav* and agonizing this pathway in WT embryos. Under physiological conditions, the PPARG pathway may act as a sensor of the balance between oxidative phosphorylation and aerobic glycolysis, and shift the balance between neural cell proliferation and differentiation accordingly.

Using *xav* as a model for MADD, we have gained new insights into the cellular and molecular mechanisms underlying this rare but devastating human disorder. *xav* is the first animal model in which the *etfdh* gene is affected and in which neural defects can be demonstrated and studied. We have also identified the PPARG-ERK pathway as potentially valuable for therapeutic intervention. Understanding the relationship between metabolic and mitochondrial deficiencies and the mechanisms underlying the pathology of MADD, in particular the neurological phenotypes, has been hampered by the rarity of the disorder and thus analyses of autopsy and other tissues has been limited[5]. It will be of particular interest to assess neural cell proliferation and other neural phenotypes in MADD patients as tissues become available. The striking phenotypic similarity between *xav* and MADD patient cells suggests that *xav* mutants will be a useful discovery tool to guide future analyses in human MADD patients, and identify avenues for therapeutic intervention.

## Supporting Information

Text S1 Supplemental Results and Methods
(0.10 MB DOC)

Table S1 Primer sequences for new zebrafish simple sequence repeat (SSR) markers.
(0.03 MB DOC)

Table S2 Primers for qRT-PCR analyses of gene expression in *xav* and fibroblasts from human MADD patients.
(0.08 MB DOC)

Figure S1 Genetic map of the *xav* locus. Conserved synteny between genes in the *xav* interval and human chromosome 4. *xav* linkage results suggest the sequence in and around BAC clone DKEY-50I13 (accession no. CR846102.12) is misplaced on the Ensembl Zv6 genome build.
(0.46 MB EPS)

Figure S2 Nonsense mediated decay and nonsense mediated alternative splicing of *etfdh* transcript in *xav*, and morpholino knock down of *etfdh*. A. qRT-PCR showed that there is a significant, ∼80% reduction of *etfdh* mRNA in *xav*, likely due to nonsense mediated decay. N = 3 pools of 20 embryos each for WT and *xav*; Student's t test, * p<0.0001. B. *xav* mutants showed nonsense mediated alternative splicing. As a result of the mutation, which resides in exon11, the exon10-exon13 junctions are mis-spliced in mutants, resulting in transcripts that are predicted to encode proteins lacking critical domains or truncated. Blue arrows indicate primer location. These results suggest that the *xav* mutation is likely to be loss of function. C. A splice-blocking morpholino against intron2-exon3 (MOI2E3) was designed for *etfdh*. D. Injection of 8 ng *etfdh* MOI2E3 in WT embryos, results in >80% reduction of the normal transcript at 2 and 3 dpf, producing a mis-spliced transcript that lacks exon3. Red arrow indicates the MO location and blue arrows indicate primer location.
(0.44 MB EPS)

Figure S3 *xav* mutants display polycystic kidney like phenotypes. Immunostaining of whole mount zebrafish embryos revealed that the cilia in the pronephric ducts, as labeled by immunostaining with anti-acetylated tubulin antibody, appear distended and irregularly thickened, and contain gaps, in *xav* mutants compared to WT embryos at ∼60 hpf. Pronephric duct epithelial cells, as labeled by immunostaining with the anti-NaK ATPase antibody α6F, appear irregular in shape and showed aberrant clustering in *xav* embryos. N = >10 xav and WT embryos for each immunostaining assessment. Scale bar = 20 µm.
(0.39 MB EPS)

Figure S4 *xav* mutants exhibit respiratory deficiency. A. Polarographic traces showing O_2_ consumption by mitochondria in WT and *xav* homogenates. Freshly prepared homogenates from WT and *xav* embryos were incubated in an oxygen sensor chamber, and O_2_ consumption (y axis) as a function of incubation time (x axis) was recorded. In the upper panels, homogenates were incubated with α-ketoglutarate + malate, and in the lower panels, homogenates were incubated with fatty acid (C16 carnitine + malate). Maximal rates of electron transfer were determined from the rates of O_2_ consumption driven by ADP (0.2 mM) and inorganic phosphate (state 3) and state 4 determined from the rate of O_2_ consumption upon conversion of the ADP to ATP. N = >10 embryos from at least 2 carrier pairs for each metabolic assay. B. At ∼56 hpf, a higher level of F1-F0 ATPase (complex V) protein was detected by immunostaining (red) in *xav* mutants (lower panels) compared to WT embryos (upper panels). A higher magnification view of the spinal cord is shown in the right most panels. TUNEL staining (green) was performed simultaneously and showed that there is increased cell death in the spinal cord in *xav* and that complex V positive cells are also TUNEL positive in *xav*. N = >10 embryos from at least 2 carrier pairs for each immunostaining assay. Scale bar = 100 µm.
(0.89 MB EPS)

Figure S5 *xav* mutants exhibit neural and glial defects and cell death. At 3 dpf, *xav* embryos exhibited increased cell death as assayed by TUNEL staining (green), reduced neuropil as assayed by SV2 staining (blue). Glia number and patterning as assayed by GFAP staining (red) was aberrant throughout the nervous system. Not only were glial processes irregular in shape, but clumps of GFAP+cells were observed in several brain regions (white arrowheads point to several examples). N>3 embryos, 1 carrier pair. Scale bar = 20 µm.
(0.33 MB EPS)

Figure S6 *xav* mutants exhibit reduced motor axon branching and neuromuscular synaptogenesis that are not caused by change of motor neuron number or viability. A. SV2 (green) and AChR (red) labeling showed that motor axon branching and neuromuscular synaptogenesis were reduced in *xav* mutants and *etfdh* morphants compared to WT embryos, at 56 hpf and most strikingly at 72 hpf. Scale bar = 20 µm. B. Motor neuron number, as assayed by *in situ* hybridization for islet-2, is similar between *xav* mutants and WT embryos at 48 hpf. Scale bar = 100 µm. C. No substantial apoptosis was seen in the pool of motor neurons in xav compared to WT at 48 hpf, as assayed by double staining for TUNEL and Zn5, which labels secondary motor neurons. N>10 embryos, 3 carrier pairs. Scale bar = 20 µm.
(0.38 MB EPS)

Figure S7 *xav* mutants display electrophysiological properties in the muscle, comparable to WT. A–C. Miniature excitatory postsynaptic current (mEPC) recordings from fast muscles showed a significant reduction in the mEPC rise time (WT 0.122±0.007 ms, xav 0.096±0.002 ms, N = 6, Student's t test, * p<0.05) and the frequency (WT 0.36±0.07 Hz, xav 0.10±0.05 Hz, N = 6; Student's t test, * p<0.05), but no statistical difference in terms of amplitude and exponential decay time. N = 6–8 embryos, 2 carrier pairs. D. Muscle fibers from *xav* embryos fire action potentials after exogenous stimulation, comparable in amplitude and shape to action potentials recorded from muscle fibers from WT embryos. There is no significant difference in terms of action potential amplitude, threshold or half width. N = 5 embryos, 2 carrier pairs.
(4.62 MB EPS)

Figure S8 *xav* mutants exhibit aberrant mitochondria distribution in motor neurons. A–B. Mito-GFP was expressed in motor neurons and branching and synapse formation followed over time. In WT embryos at ∼56 hpf, mitochondria are distributed along the entire axon. Mitochondria are continuously added over time, increasing both in number and density. In contrast, in *xav* mutants, while the number and distribution of mitochondria clusters along the axon are comparable to WT embryos at ∼56 hpf, the continuous addition of mitochondria is absent, resulting in a decrease of mitochondria cluster density by 3 dpf. N = 9–10 embryos, 3–4 carrier pairs. Scale bar = 20 µm.
(0.51 MB EPS)

Figure S9 *xav* mutants exhibit cell death throughout the nervous system that is rescued by p53 morpholino knockdown and does not account for the motor axon branching, neuromuscular synapse or motility defects. A. *xav* mutants exhibit widespread cell death in the peripheral and central nervous system. There was a dramatic increase in TUNEL+cells in *xav* mutants compared to WT embryos at ∼56–72 hpf, particularly in the retina and spinal cord. B. At 56 hpf, cell death was blocked using a morpholino against p53. However, blocking cell death did not block the reduction of axon branching and synaptogenesis phenotypes that are present in *xav* mutants. N>6 embryos, 2 carrier pairs. Scale bar = 100 µm.
(0.39 MB EPS)

Video S1 Spontaneous movements and motility in wild type zebrafish embryos at 48 hpf. The motility of wild type and *xav* mutant embryos were examined using high-speed video microscopy at 48 hpf. Wild type embryos spend most of the time staying still when not stimulated, and exhibit quick, darting movements when they swim.
(3.98 MB AVI)

Video S2 *xav* mutants exhibit increased spontaneous movement but with reduced motility at 48 hpf. The motility of wild type and *xav* mutant embryos were examined using high-speed video microscopy at 48 hpf. *xav* mutants show increased spontaneous twitching when not stimulated, and exhibit reduced motility when they swim, often exhibiting spasmodic like movements.
(4.91 MB AVI)
