# Supplementary material for: Mechanisms Underlying Metabolic and Neural Defects in Zebrafish and Human Multiple Acyl-CoA Dehydrogenase Deficiency (MADD)
Source: PLoS One. 2009 Dec 17;4(12):e8329. doi: 10.1371/journal.pone.0008329 (PMC2791221; doi:10.1371/journal.pone.0008329)
Supplement: Text S1 — Supplemental Results and Methods (0.10 MB DOC) [file pone.0008329.s001.doc]

**SUPPLEMENTAL INFORMATION**

**Supplemental Materials and Methods**

**Positional cloning of *xav***

Adult zebrafish heterozygous for the *xav* mutation were crossed to generate a panel for linkage mapping. DNA was isolated from *xav* homozygous mutant embryos, wild type siblings, and parental fin clips. Bulk segregant analysis was performed as described[7], using microsatellite markers[8,9] (http://zebrafish.mgh.harvard.edu/zebrafish/index.htm). Once linkage was detected, 96 mutant individuals were genotyped to localize the mutation between two closely flanking markers. These analyses mapped *xav* to chromosome 14 between markers Z15804 and Z7108. An additional 749 *xav* mutant embryos were collected from two mapping crosses and used for further linkage analysis. New simple sequence repeat (SSR) markers were identified from sequenced BAC clones and primers were designed using the zebrafish SSR search web site (<http://danio.mgh.harvard.edu/markers/ssr.html>). Primer sequences for new markers are given in Supplemental Table 1. PCR reactions were run with 5 PRIME Taq DNA polymerase according to the manufacturer’s instructions. Genotypes were determined by analyses of PCR products on 4% agarose E-Gels (Invitrogen). For genotyping large numbers of fish we used fluorescently labeled primers and analyzed products on ABI 3130xl Genetic Analyzer (Applied Biosystems).

To sequence the open reading frame (ORF) of candidate genes, RNA was isolated from pools of mutant and WT embryos using a VERSAGENE RNA isolation Tissue Kit (Gentra Systems). cDNA was synthesized using Oligo (dT) and Superscript III as described by the manufacturer (Invitrogen). cDNA fragments of the entire ORF were obtained by PCR using Accuprime Taq polymerase and Buffer II (Invitrogen), 20mM of each primer (Sup. Table 1) and first strand cDNA. Amplified products were purified and sequenced using the ABI Big dye terminators and an ABI 3130xl Genetic Analyzer. When necessary, PCR products were cloned into pCR 4-TOPO (Invitrogen), plasmid DNA isolated with Wizard Plus SV Minipreps (Promega) and inserts sequenced as described above.

**Polarographic analysis of oxygen consumption, mitochondrial membrane potential measurement, ATP and lactate quantification**

WT and *xav* embryos (50-100 embryos for each sample) were homogenized in a mitochondrial isolation buffer (MIB) (in mM, 210 mannitol, 70 sucrose, 10 HEPES (pH 7.2), 0.2 EGTA) freshly supplemented with 0.5% fatty acid- free BSA and 1/100 dilution of Sigma protease inhibitor cocktail. All operations and buffers were at 2-4^o^C.

Oxygen consumption was measured polarographically using a Strathkelvin oxygen electrode system at 28°C. Homogenates were suspended in a total volume of 0.15 mL of air-saturated buffer composed (in mM) of 220 mannitol, 70 sucrose, 5 HEPES, pH 7.2, 5 KH2PO4, 0.2 EGTA and 5 EDTA. Oxidation of carnitine esters was measured in the presence of 2 mM malate with either 0.10 mM palmitoylcarnitine or 0.2 mM octoylcarnitine followed by addition of 0.2 mM ADP. Oxygen consumption was also measured in the presence of 10 mM α-ketoglutarate + 2 mM malate or 10 mM succinate + 2 µM rotenone followed by addition of 0.2 mM ADP. Azide-sensitive cytochrome oxidase activity (complex IV) was measured in the presence of 2.5 mM ascorbate + 0,25 mM N,N,N′,N′-tetramethyl-p -phenylenediamine (TMPD). Rates of substrate oxidation with or without ADP were expressed as nanoatoms of oxygen consumed per minute per milligram homogenate protein. State 3 refers to oxygen consumption stimulated by a limiting amount of ADP and state 4 refers to oxygen consumption after phosphorylation of the added ADP to ATP.

Tetra-methyl rhodamine ester (TMRE, Invitrogen) was used to measure generation of mitochondrial transmembrane potential. Used in the quench mode, energization of mitochondria decreases and uncoupling increases TMRE fluorescence measured using excitation/emission wavelengths of 549 nm and 574 nm, respectively, in an Aminco-Bowman Series 2 spectrofluorometer. Homogenates were suspended in buffer composed (in mM) of 220 mannitol, 70 sucrose, 10 Hepes, pH 7.2, 5 KH2PO4, 0.2 EGTA and 200 nM TMRE. Fluorescence changes were monitored continuously while the sample was magnetically stirred at ambient temperature. Energization was achieved by addition of 2 mM malate with 0.10 mM palmitoylcarnitine or 0.2 mM octoylcarnitine, or 10 mM α-ketoglutamate + 2 mM malate; mitochondria were depolarized by addition of 5 µM ClCCP.

Measurement of intact cellular respiration was performed using the Seahorse XF24 analyser[10]. Respiration was measured under basal condition, in the presence of the mitochondrial ATP synthase inhibitor oligomycin (0.5 μg/ml), in the presence of the mitochondrial uncoupler carbonyl cyanide *m*-chlorophenylhydrazone (CICCP) (3 μM) to maximally stimulate respiration and in the presence of complex I inhibitor rotenone (100 nM) to assess residual non-mitochondrial respiration as described previously.

For ATP quantification, fibroblasts or embryo homogenate were prepared in MIB and all operations performed at 4°C. While continuously vortexing, 0.1 volume of 6 N PCA was added to homogenates while vortexing to achieve a final concentration of 0.6 N PCA. Following a 15 min incubation on ice to facilitate full precipitation, samples were centrifuged at 15,000 g for 15 min. Extracts were transferred to a clean tube and sufficient 2M KHCO3 was added while vortexing to achieve a final concentration of 0.5M KHCO3. Extracts were centrifuged at 15,000 g for 15 min, transferred to a clean tube and frozen at -80°C until assayed using a ATP luminescence kit (Sigma). Luminescence was read using a Turner luminometer. Protein concentration was quantified using the BCA assay (Pierce) with BSA as a standard. For lactate measurement in fibroblasts, culture media was collected after 6 days *in vitro* and assayed with Lactate Assay Kit (Biovision) and a fluorometer. For lactate measurement in embryos, embryos extracts were prepared as for ATP measurement, and were assayed using Lactate Assay Kit (Biovision) by measuring absorbance at 450 nm with a microplate reader.

**Behavioral assessment of *xav* mutants and wild type embryos**

Gross motility was assessed via head and/or tail tap with a fine plastic probe, and elicited movements observed under a stereomicroscope (Leica MZ12.5). The behavior was recorded using high-speed video microscopy (800 frames/ s; MotionPro 2000, Redlake), Supplemental Video 1 and 2.

**Immunostaining and TUNEL assay**

Embryos were anesthetized, fixed and immunostained as described previously [3] using antibodies against the presynaptic neurotransmitter vesicle protein SV2 (Developmental Studies Hybridoma Bank (DSHB)), Zn5 (DSHB) and/or the glial specific protein GFAP[11] (gift from Drs. S. Nona and J. Scholes, Univ. of Sussex, United Kingdom), complex V (Invitrogen) and the appropriate fluorescently conjugated secondary antibody (Jackson Labs). To label AChRs, fluorescently conjugated α−bungarotoxin (Invitrogen) was used as described previously[3]. Presynaptic vesicles, AChR clusters and the co-localization of these two markers were measured from single plane projections of confocal image stacks using interactive software (Metamorph). For immunostaining of NaK ATPase with α6F (DHSB) together with Acetyl-tub (Sigma), embryos were fixed in Dent’s fix[12]. For sections, fixed embryos were incubated in 20% sucrose, embedded in OCT and cut into 20 μm sections.

TUNEL staining was performed according to the manufacturer’s instructions (Chemicon).

**p53 morpholino injection**

A morpholino antisense oligonucleotide (Gene Tools) targeting p53 (GCGCCATTGCTTTGCAAGAATTG)[5] was injected at the 1-2 cell stage at ~8 ng. Embryos were assessed at 56-72 hpf using the TUNEL assay.

**Mitochondria labeling in motor neurons**

An *hb9*:mito-GFP construct was generated by inserting mito-GFP (obtained from Drs. Marnie Halpern and Bill Saxton, Carnegie Inst. for Dev. Biology, Baltimore, MD) into an I-Sce vector containing *hb9* promoter (obtained from Dr. Michael Granato, Univ. of Pennsylvania Sch. Medicine and Dr. Dirk Meyer, Albert-Ludwigs University, Freiburg, Germany). DNA (50 ng/μl) was injected into 1-2 cell stage embryos, which were then raised to 24-48 hpf for assessing expression in motor neurons using confocal microscopy.

**Muscle fiber electrophysiology**

Miniature endplate currents were recorded from the fast muscle fibers of wild type *and xav* embryos at approximately 56-72 hpf. Embryos were anesthetized, pinned through the notochord to Sylgard-lined glass slides, and perfused with extracellular saline containing (in mM) 134 NaCl, 2.9 KCl, 2.1 CaCl, 1.2 MgCl_2_, 10 HEPES, 10 glucose and 0.001 tetrodotoxin (TTX, Tocris, Avonmouth UK). The nerve cord at the junction of the spinal cord and the hindbrain was pinched and severed with a fine pair of forceps. A section of the skin overlaying the trunk was removed to allow access to axial muscles. Whole cell patch clamp recordings were performed on axial fast fibers in voltage clamp mode[13]. The pipette solution contained (in mM) 130 CsCl, 2 NaCl, 10 HEPES, 10 EGA, 2 CaCl_2_, 4 MgATP and 0.4 LiGTP. Polished pipettes were pulled from thin walled glass (World Precision Instruments, Sarasota Fl, USA) and had tip resistances of 1.0-1.8 MΩ resulting in whole cell series resistance values of 1.6-3.6 MΩ. Series resistances were monitored carefully and the recordings were abandoned if they changed by 15% or greater. All series resistances were compensated by 90% and fibers were voltage clamped at -60 mV throughout the recordings. Recordings were performed with an Axopatch 200B and mEPCs were captured with pClamp 8.1 software. All mEPCs were analyzed offline with Axograph X software. Data were sampled at 50 KHz and low-pass filtered at 10 KHz.

Action potentials were recorded in current-clamp mode where the extracellular saline consisted of (in mM) 124 NaCl, 2.9 KCl, 0.7 CaCl_2_, 10 MgCl_2_, 10 HEPES and 10 Glucose. The pipette-filling solution consisted of (in mM) 140 D-gluconic acid K^+^ salt, 6 KCl, 8 NaCl, 10 EGTA, 10 HEPES, 4 MgATP and 0.4 LiGTP. A short current pulse, 1 ms in duration, was injected into the muscle fiber to bring the cell to threshold. All solutions and drugs were bath-applied at a flow rate of 1-2 ml/min. All drugs were acquired from Sigma (St Louis, MO), unless otherwise indicated.

**Supplemental Results**

**Positional cloning of *xav* mutation**

Positional cloning strategies were used to identify the mutation responsible for the *xav* phenotype. Bulk segregant analysis initially localized the mutation to chromosome 14 between Z15804 and Z7108 on linkage group 14. Further mapping using newly designed SSR markers localized the mutation between 274P15-SSR1 and 199B20-SSR1 (Figure 1C). Sequence from the Ensemble Zv6 genome build did not appear to be correctly ordered between the flanking markers as was evidenced by the fact that our linkage data indicated markers 36H4-SSR1 (Figure 1B) and Z4701 (data not shown) fell outside the interval while the Zv6 build placed these sequences directly between the flanking markers (data not shown). For this reason, we asked if the general order of some of the genes in the region could be confirmed based on conserved synteny with human. We found that *LOC557693*, *tmem33*, *zgc:92093*, and *ppid* potentially represented a conserved block of genes (Figure S1). SSR markers were then designed in BAC clones that contained these genes to determine if the sequences were truly positioned within the interval. Following this strategy, marker 278P11-SSR1 was found to have zero recombinants. Interestingly, the linkage results also indicated that BAC DKEY-50I13, positioned roughly 50 Mb from this region on the Zv6 build, was in fact located in the critical interval (Figure 1C). The open reading frames for the three genes in BAC CH211-278P11 (*LOC565292*= *rxfp1-like*, *zgc:92093*, and *ppid*) were amplified from mutant and wild-type embryos and sequenced to look for mutations. *zgc:92093* was found to contain a T to A transversion at position 1305 which introduces a premature stop codon (Y435X) in the 617 amino acid protein (Figure 1D, 1E). *zgc:92093* encodes electron-transfer-flavoprotein dehydrogenase (etfdh) which is highly conserved among species and is 80% homologous to its human counterpart.

***Kidney phenotypes in xav mutants***

Given that congenital polycystic kidneys have been reported in MADD patients[1,2], we asked whether *xav* mutants display similar phenotypes. Immunostaining of whole mount embryos at ~60 hpf revealed that the cilia in the pronephric ducts, as stained by anti-acetylated tubulin, appear distended and irregularly thickened, and contain gaps, in *xav* mutants compared to WT embryos (Figure S3). Pronephric duct epithelial cells, as labeled by an anti-NaK ATPase antibody, appear irregular in shape and showed aberrant clustering in *xav* embryos (Figure S3). This result suggests that *xav* exhibit kidney morphological defects similar to those in MADD patients.

***xav mutants exhibit abnormal motility and progressive paralysis***

*xav* mutants exhibited several behavioral deficits. While *xav* mutant embryos are normally motile up until 48 hpf, at this time, increased spontaneous muscle twitching and spastic movements are observed in mutants, while WT embryos are largely stationary unless stimulated (percent of time spent continuously moving, WT 0.4% ± 0.3%, *xav* 12% ± 4%, N = 3-5 embryos, 1 carrier pair, Student’s t test, p<0.05) (Supplemental Video 1 and 2). However, once stimulated, WT embryos respond with a characteristic C-bend and swimming escape response[3], but *xav* mutants, despite executing a C-bend similar to that in WT embryos, do not exhibit a normal swimming escape response. *xav* mutants swim with reduced speed and distance, often ending in twitching movements (Supplemental Video 1 and 2). Moreover, by ~60 hpf, *xav* mutants are completely paralyzed[3].

***xav mutants exhibit several neural phenotypes, including reduced neuropil staining and aberrant glial patterning***

Several analyses were performed to qualitatively assess the central and peripheral nervous system in *xav* mutants compared to WT embryos, to further understand the neural defects in *xav*, and provide insight into those neural defects that may be present in MADD patients, as this is a poorly explored aspect of the disorder due to its rarity[4].

At 3 dpf, *xav* embryos exhibited reduced neuropil as assayed by SV2 staining (Figure S5). Glia number and patterning as assayed by GFAP staining was aberrant throughout the nervous system (Figure S5). Not only were glial processes irregular in shape, but clumps of GFAP+ cells were observed in several brain regions (Figure S5 and data not shown). These observations suggest that, in addition to neural cell proliferation defects, several aspects of neural development are impaired in *xav*, due to metabolic and mitochondria deficiency.

***xav mutants exhibit reduced motor axon branching, reduced neuromuscular synapse number and abnormal mitochondria distribution within motor neuron axons and at synapses***

*xav* mutants exhibit reduction of motor axon branching, and a reduction of neuromuscular synaptogenesis, at 56 hpf and most strikingly at 72 hpf (Figure S6A; and[3]). Motor neuron number, as assayed by in situ hybridization for *islet-2*, is similar between *xav* mutants and WT embryos at 48 hpf (Figure S6B). Moreover, there is no substantial apoptosis in the pool of motor neurons, as assayed by double staining for TUNEL and Zn5, which labels secondary motor neurons (Figure S6C). These data suggest that the reduction in motor axon branching and neuromuscular synaptogenesis are not due to a decrease in motor neuron number.

To determine whether motor neurons and muscle fibers function normally in *xav* mutants, electrophysiological assays were performed in *xav* mutants and WT embryos. Miniature excitatory postsynaptic current (mEPC) recordings from fast muscles showed significant differences in the mEPC rise time and frequency, but no statistical difference in terms of mEPC amplitude and exponential decay time (Figure S7A, 7B, 7C). These findings are consistent with a reduced number of neuromuscular synaptic sites in *xav* mutants compared to WT embryos, ascertained via immunohistochemistry. Furthermore, muscle fibers from *xav* embryos fire action potentials after exogenous stimulation, comparable to muscle fibers from WT embryos (Figure S7D). These data suggest that despite a reduction of neuromuscular synapses in *xav* mutants, the overall physiological properties of postsynaptic muscle fibers are not compromised, making the possibility of deteriorative and nonspecific effects in muscle fibers an unlikely explanation for the morphological and motility defects. Thus, either defects intrinsic to presynaptic motor neurons, and/or circuitry in the spinal cord and/or other CNS regions, is responsible for the observed paralysis in *xav* mutants.

We next assessed the distribution of mitochondria within motor neurons. Mito-GFP was expressed in motor neurons and branching and synapse formation followed over time. In WT embryos at ~56 hpf, mitochondria are distributed along the entire axon and some appear to be clustered (Figure S8A). The majority of mitochondria clusters are at synapses, with some along the axon and branches (data not shown). Mitochondria clusters are continuously added over time, increasing both in number and density (Figure S8A, 8B). In contrast, in *xav* mutants, while the number and distribution of mitochondria clusters along the axon are comparable to WT embryos at ~56 hpf, the continuous addition of mitochondria is absent, resulting in a decrease of mitochondria cluster density over time (Figure S8A, 8B). These results suggest that the number, distribution and addition of mitochondria is impaired in *xav* mutants.

***xav mutants exhibit cell death throughout the nervous system that is rescued by p53 morpholino knockdown and does not account for the motor axon branching, neuromuscular synapse or motility defects***

A particularly prominent phenotype in *xav* mutants is the widespread cell death observed in the peripheral nervous system, and most strikingly in the CNS, compared to other tissues. There was a dramatic increase in TUNEL+ cells in *xav* mutants compared to WT embryos at ~56-72 hpf, particularly in the retina and spinal cord (Figure S9A). That TUNEL+ cells were not observed in all tissues argues against generalized cell death due to metabolic insufficiency.

Given prominent apoptotic cell death throughout the nervous system, we asked whether blocking cell death may abrogate any *xav* neural and behavioral phenotypes. Cell death was blocked using a morpholino against p53[5,6] and *xav* and WT embryos were assessed at ~56 hpf. *xav* mutants injected with p53 morpholino exhibited levels of cell death that were indistinguishable from WT in the central and peripheral nervous system (Figure S9B). However, blocking cell death did not block the reduction of axon branching and synaptogenesis phenotypes that are present in *xav* mutants, as these phenotypes were similar between *xav* mutants and *xav* mutants injected with p53 morpholino (Figure S9B). Moreover, *xav* mutants injected with p53 morpholino exhibited motility deficits that were indistinguishable from *xav* mutants, including the proportion of embryos paralyzed at ~56-60 hpf (data not shown). This result suggests that cell death per se is unlikely to account for the axon branching, synaptogenesis or paralysis phenotypes prominent in *xav* mutants.

**Supplemental References**

1. Bohm N, Uy J, Kiessling M, Lehnert W (1982) Multiple acyl-CoA dehydrogenation deficiency (glutaric aciduria type II), congenital polycystic kidneys, and symmetric warty dysplasia of the cerebral cortex in two newborn brothers. II. Morphology and pathogenesis. Eur J Pediatr 139: 60-65.

2. Lehnert W, Wendel U, Lindenmaier S, Bohm N (1982) Multiple acyl-CoA dehydrogenation deficiency (glutaric aciduria type II), congenital polycystic kidneys, and symmetric warty dysplasia of the cerebral cortex in two brothers. I. Clinical, metabolical, and biochemical findings. Eur J Pediatr 139: 56-59.

3. Panzer JA, Gibbs SM, Dosch R, Wagner D, Mullins MC, et al. (2005) Neuromuscular synaptogenesis in wild-type and mutant zebrafish. Dev Biol 285: 340-357.

4. Frerman FE, Goodman SI (2001) Defects of electron transfer flavoprotein and electron transfer flavoprotein ubiquinone oxidoreductase: glutaric acidemia type II. In: Scriver CR BA, Sly WS, Valle D, editor. The metabolic and molecular bases of inherited diseases. New York: McGraw-Hill. pp. 2357-2365.

5. Langheinrich U, Hennen E, Stott G, Vacun G (2002) Zebrafish as a model organism for the identification and characterization of drugs and genes affecting p53 signaling. Curr Biol 12: 2023-2028.

6. Robu ME, Larson JD, Nasevicius A, Beiraghi S, Brenner C, et al. (2007) p53 activation by knockdown technologies. PLoS Genet 3: e78.

7. Willer GB, Lee VM, Gregg RG, Link BA (2005) Analysis of the Zebrafish perplexed mutation reveals tissue-specific roles for de novo pyrimidine synthesis during development. Genetics 170: 1827-1837.

8. Knapik EW, Goodman A, Ekker M, Chevrette M, Delgado J, et al. (1998) A microsatellite genetic linkage map for zebrafish (Danio rerio). Nat Genet 18: 338-343.

9. Shimoda N, Knapik EW, Ziniti J, Sim C, Yamada E, et al. (1999) Zebrafish genetic map with 2000 microsatellite markers. Genomics 58: 219-232.

10. Ferrick DA, Neilson A, Beeson C (2008) Advances in measuring cellular bioenergetics using extracellular flux. Drug Discov Today 13: 268-274.

11. Nona SN, Shehab SA, Stafford CA, Cronly-Dillon JR (1989) Glial fibrillary acidic protein (GFAP) from goldfish: its localisation in visual pathway. Glia 2: 189-200.

12. Drummond IA, Majumdar A, Hentschel H, Elger M, Solnica-Krezel L, et al. (1998) Early development of the zebrafish pronephros and analysis of mutations affecting pronephric function. Development 125: 4655-4667.

13. Hamill OP, Marty A, Neher E, Sakmann B, Sigworth FJ (1981) Improved patch-clamp techniques for high-resolution current recording from cells and cell-free membrane patches. Pflugers Arch 391: 85-100.
