# Supplementary material for: Mechanisms Underlying Metabolic and Neural Defects in Zebrafish and Human Multiple Acyl-CoA Dehydrogenase Deficiency (MADD)
Source: PLoS One. 2009 Dec 17;4(12):e8329. doi: 10.1371/journal.pone.0008329 (PMC2791221; doi:10.1371/journal.pone.0008329)
Supplement: Table S1 — Primer sequences for new zebrafish simple sequence repeat (SSR) markers. (0.03 MB DOC) [file pone.0008329.s002.doc]

**Supplemental Table 1.** Primer sequences for new zebrafish simple sequence repeat (SSR) markers

| **SSR Name** | **Forward** | **Reverse** |
| --- | --- | --- |
| Z15804-SSR4 | AACATGTTTGGGTATCCTGATTT | TGACCACCAGAATCAAGACG |
| 274P15-SSR1 | GGAAGCTAATTGCACGGTCT | AACCCACTGATAACAGTTCCAA |
| 50I13-SSR1 | TGCGTGCAATCAGGTTTAAG | TGTCCCAGTGACCAACATGA |
| 278P11-SSR1 | ATGGCAGCCTTCACCAAACA | CACCCGGAGTTCACCTTTCA |
| 276B5-SSR3 | TGCTTGCTAAGGTATTTGCTGA | TTAAAGCAGCAAGGCAATGA |
| 154C1-SSR1 | CAGGTATGAAATGCTGCTTGC | TTGCTCCAGCAGAGATGATG |
| 199B20-SSR1 | AGCTCCCAGGATTCCACTCT | CCCTAACTGTCTCTGCAACCA |
| 36H4-SSR1 | ATCCCTTTTGGGAAAACAGC | ACCTCATCACGTACGCACAA |
| **Gene Name** |  | |
| *zgc:92093* (Acc # NM_001004598) | CGCATTCAACCTGGAAAACC | TGTGCCCCAGTGACGGTAAT |
