# Supplementary material for: Mechanisms Underlying Metabolic and Neural Defects in Zebrafish and Human Multiple Acyl-CoA Dehydrogenase Deficiency (MADD)
Source: PLoS One. 2009 Dec 17;4(12):e8329. doi: 10.1371/journal.pone.0008329 (PMC2791221; doi:10.1371/journal.pone.0008329)
Supplement: Table S2 — Primers for qRT-PCR analyses of gene expression in xav and fibroblasts from human MADD patients. (0.08 MB DOC) [file pone.0008329.s003.doc]

**Supplemental Table 2.** Primers for qRT-PCR analyses of gene expression in *xav* and fibroblasts from human MADD patients

| **Gene Name** | **Forward** | **Reverse** |
| --- | --- | --- |
| **Zebrafish** | | |
| *etfdh* | acacgctggttctcctctgt | agcgaagcgttccatttcta |
| *β-actin1* | TGAATCCCAAAGCCAACAGAGAGA | TCACGACCAGCTAGATCCAGACG |
| *mt-nd5* | TTGACCATCGGGAAAATAGC | TTAGGGCTCAGGCGTTAAGA |
| *ndufs1* | gccgtatgacacactggatg | ctcatgggcctgtttgaaat |
| *pgc-1β* | GGACGCCAGTGATTTTGACT | TCCAGCGCTGTACTGTATGG |
| *esrra* | cgaggagtttgtcatgctca | ctgatgcagcaggtctcgta |
| *pparγ* | AGTACGGGGTCATCGAAGTG | GCGCAGACTCTTGAGGAACT |
| *ucp4* | gcgtggaaaagacaacccta | aaacgcacctttaccacgtc |
| *ndufb8* | tctcctgaccccagctctaa | ctttctcctctgcggttttg |
| *cox5aa* | acggatgaggagtttgatgc | tccaggatctttggttcagg |
| *atp5g* | aggaacccatctctgaagca | gcaaacaggatgaggaaagc |
| *ndufv1* | cttgcagagagggagtggac | gtctcctaaagcgcagatgg |
| *pgc-1α* | ctgcctgagcttgacctttc | ttggatgcttcattgccata |
| *nrf1* | ccgaacagaggagcagaaac | gtggcaacttgtgtggtgac |
| *tfam* | agctggcagaggacgataaa | gttttggctttggctttgag |
| *pparα* | cgacaagtgtgaacgcaact | tatccgcccaaaacgaatag |
| *pparβ* | tggagtacgagcgatgtgag | cttctccgcttctggcatac |
| *ucp2* | agctggtgacgttcctccta | ttgttctccccctgaatctg |
| *cyt C* | gcattgtctggggtgaagat | tctctcgcccttcttcttga |
| *catalase* | gcggataccagagagagtcg | atcggtgtcgtctttccaac |
| *glutathione reductase* | attggcagagaacccaacac | acatccccgactgcatagac |
| *gpx1a* | gaaatacgtccgtcctggaa | tctcccataagggacacagg |
| *gpx3* | TCCAGGAAATGGATTCGTTC | TCTCTCCTACAGGCGGACAT |
| *hmox1* | ggaagagctggacagaaacg | cgaagaagtgctccaagtcc |
| *hspa9* | cgacttgggaaccacaaact | cattcctacaagccgctctc |
| *eno1* | ggcaaaggtgtctcaaaagc | ttgttgtctgtgccatccat |
| *pgam1a* | tgagaggcattgtgaagcac | ggcttcaggttcttgtccag |
| *pgk1* | gatggagtccctatgccaga | tggacccacacagtctttca |
| *pfkma* | ctggaagccactccagagac | aatcgaccctcattcattgc |
| *aldoc* | ccattgtggagcctgagatt | tcaccatgttgggtttcaga |
| *dlat* | gagacctgctggctgaaatc | ctggtgccctcagaaatcat |
|  | | |
| **Human fibroblasts** | | |
| *ACTB* | GAGCTACGAGCTGCCTGACG | GTAGTTTCGTGGATGCCACAG |
| *PGC-1β* | ttatgcctccctcacacctc | tgaagctgcgatccttacct |
| *PPARG* | gagcccaagtttgagtttgc | ggcggtctccactgagaata |
| *ESRRα* | AAAGTGCTGGCCCATTTCTAT | CCTTGCCTCAGTCCATCAT |
| *UCP3* | ctccaggccagtacttcagc | cgcaaaaaggagggtgtaaa |
| *catalase* | TTTCCCAGGAAGATCCTGAC | ACCTTGGTGAGATCGAATGG |
| *glutathioine reductase* | acttgcccatcgactttttg | catcttccgtgagtcccact |
| *HSPA9* | aattacttggggcacacagc | cgaagcacattcagtccaga |
| *ENO1* | ctccgtgaccgagtctcttc | ccagtcttgatctgcccagt |
| *PGAM1* | ggggtctaaccggtctcaat | acgtttcccctccttgatct |
| *PGK1* | tcactcgggctaagcagatt | cagtgctcacatggctgact |
| *PFKM* | agagcgtttcgatgatgctt | gttgtaggcagctcggagtc |
